# Analysis of trends in the spectrum of oral diseases at the Chinese level‑II hospital for peacekeeping: A case study from the Democratic Republic of the Congo

**DOI:** 10.1371/journal.pone.0358265

**Published:** 2026-09-15

**Authors:** Yifan Zhang, Juanqin Niu, Gang Chen, Dong Qin, Xiangjie Li, Lei Xu, Zipeng Li, Wei Xue

**Affiliations:** 1 The 940th Hospital of Joint Logistic Support Force of Chinese People’s Liberation Army, Lanzhou, Gansu Province, China; 2 General Hospital of Western Theater Command, Chengdu, Sichuan Province, China; 3 Center for Disease Control and Prevention in Western Theater Command, Lanzhou, Gansu Province, China; University of Puthisastra, CAMBODIA

## Abstract

**Background:**

Oral diseases represent an important reason for consultation among personnel attending the Chinese level-II hospital in the Democratic Republic of the Congo (DRC). By analyzing the trends in the spectrum of oral diseases, we aim to improve precision support in oral healthcare for peacekeepers.

**Materials and methods:**

Clinical data from outpatient visits during Batches 23, 25, and 26 at the hospital were collected. A total of 565 oral outpatient visits (205 in Batch 23, 154 in Batch 25, and 206 in Batch 26) were analyzed. Oral diseases were classified using ICD-11 into five categories, and their proportions and trends were compared using chi‑square tests and Cochran−Armitage trend tests with Bonferroni correction.

**Results:**

Oral outpatient visits accounted for the largest proportion of specialized outpatient consultations across all three batches, accounting for 22.19% (Batch 23), 21.21% (Batch 25), and 25.40% (Batch 26) of all outpatient visits. The proportion of diseases of hard tissues of teeth increased from 29.27% (Batch 23) to 49.03% (Batch 26) (*P* for trend < 0.001), and was significantly higher in Batch 26 than in Batches 23 and 25 (*P* < 0.001), with caries being the most common. Caries proportion rose from 11.71% (Batch 23) to 30.10% (Batch 26) (*P* for trend < 0.001). Among diseases of pulp or periapical tissues, pulpitis was the most prevalent; its proportion peaked in Batch 25 (33.12%) and then significantly decreased in Batch 26 (20.87%, *P* = 0.009). Periodontal disease declined from 15.12% (Batch 23) to 6.80% (Batch 26) (*P* for trend = 0.008), with Batch 26 significantly lower than Batch 23.

**Conclusion:**

This study provides preliminary insights for oral medical support in peacekeeping missions with similar settings, ensuring effective oral health protection for peacekeepers.

## Introduction

MONUSCO, the United Nations Stabilization Mission in the DRC, is authorized to take all necessary means to protect civilians and support peace consolidation efforts in the country [1]. The Chinese level‑II hospital, subordinate to the MONUSCO Southern Sector, provides medical support to military and civilian peacekeeping personnel from multiple countries [2]. Its stomatology department is a critical component, staffed by one dentist and one dental assistant in accordance with United Nations level‑II requirements [3]. In the eastern DRC, access to oral healthcare is extremely limited, and local dental resources are markedly inadequate. Consequently, the local healthcare system cannot provide substantive support to the Chinese level‑II hospital in terms of dental supplies, equipment maintenance, or referral services. This makes it essential for the hospital to rely on its own data to optimize resource allocation and to anticipate the spectrum of oral diseases among the peacekeeping personnel it serves.

Oral diseases account for a high proportion among outpatient visits in multiple peacekeeping mission areas [4,5]. Our data reveal that since September 2019, oral outpatient visits at the Chinese level-II hospital have consistently ranked first in number and proportion among all specialized outpatient services, posing a severe threat to the physical health of peacekeepers and presenting significant challenges to oral healthcare delivery at the facility. MONUSCO personnel served by the hospital are deployed within the southern sector. The southern sector’s area of responsibility covers the southern part of South Kivu Province, including Bukavu, Uvira, and surrounding areas. Since 2018, MONUSCO has been gradually withdrawing its forces from the DRC. Consequently, the total number of personnel eligible for care at the Chinese level‑II hospital gradually decreased from approximately 6,000 [4].

Despite this high burden, there is a lack of longitudinal data on the spectrum of oral diseases and their trends over time in peacekeeping settings. Peacekeepers represent a unique population due to their exposure to high-stress environments, limited access to routine dental care, reliance on the Chinese level-II hospital services, and the logistical challenges of resupplying medical materials in remote mission areas. Existing studies have focused on single time periods, but no study has systematically analyzed how the distribution of oral diseases evolves across consecutive deployment batches in the same mission area. This gap hinders evidence-based allocation of dental supplies and personnel for future rotations.

This study aimed to answer the following question: how does the spectrum of oral diseases change across three consecutive batches (Batch 23, Batch 25, and Batch 26) at the Chinese level-II hospital in the DRC. The primary outcomes were the consultation-based proportions of five oral disease categories across the three batches and the trends in selected common diagnoses (caries, pulpitis, periapical periodontitis, and gingivitis). The secondary objective was to derive operational implications for future dental resource allocation in similar peacekeeping settings.

Consequently, this study retrospectively summarizes the disease spectrum of patients attending the oral outpatient clinic at the Chinese level-II hospital in the DRC over three batches spanning October 2019 to September 2020 (Batch 23), October 2021 to September 2022 (Batch 25), and October 2022 to September 2023 (Batch 26). It analyzes the types of common and frequently occurring oral diseases in the mission area and their trends in proportions. Given the limited medical supplies in peacekeeping mission areas and the difficulties in replenishing them, this study aims to provide further insights for future peacekeeping medical contingents deployed to Africa or other regions with similar settings to more accurately and reasonably allocate corresponding medical resources. Ultimately, it seeks to enhance the diagnostic and treatment capabilities of oral specialized outpatient services at the Chinese level-II hospital, thereby effectively safeguarding the oral health of personnel in mission areas.

## Materials and methods

### Data collection

Data were collected from the outpatient case records of the Chinese level-II hospital in the DRC for Batch 23 (covering 1 October 2019–30 September 2020), Batch 25 (covering 1 October 2021–30 September 2022), and Batch 26 (covering 1 October 2022–30 September 2023). All oral outpatient visits during the study periods of these batches were included. Batches 23, 25, and 26 represent all consecutive deployments for which complete and reliable clinical records were available at the time of data collection. Batch 24 was excluded in its entirety due to incomplete records. No missing data were present for the variables analyzed (diagnosis, age, and sex), as all data were extracted from complete clinical records; incomplete batches were excluded entirely rather than imputed. Data for this study were accessed on 10 February 2025 for research purposes. The dataset used for this retrospective analysis was fully de-identified prior to being provided to the research team. The personnel composition includes: (1) Uniformed personnel: peacekeeping troops, military observers, staff officers, and formed police units. (2) Non-uniformed personnel: United Nations civilian personnel within the mission area, composed of non-military and non-police personnel from multiple countries. The hospital does not provide services to the local Congolese population. Exclusion criteria: (1) Batch 24 was excluded in its entirety because its medical records were incomplete. (2) patients who repeatedly visited for the same disease (27 in Batch 23, 30 in Batch 25, and 17 in Batch 26) were excluded after the first visit. For repeated same-disease visits, only the first visit per patient was included; subsequent visits for the same disease were excluded. Data completeness: The final dataset comprises 565 observations (205 from Batch 23, 154 from Batch 25, and 206 from Batch 26), with no missing data for the analyzed variables. The anonymized dataset supporting the findings of this study is available as S1 Data.

Data were extracted independently by two trained researchers using a standardized case report form, and discrepancies were resolved by consensus with a third reviewer. No additional instruments or measurement tools were used; all data were derived from routine clinical records.

### Methods

The total number of outpatient cases, the spectrum of oral diseases, and their trends across the three batches were analyzed.

(1) Demographic information of patients: gender and age (in years).
(2) Classification of oral diseases: Oral diseases were classified into five categories based on the International Classification of Diseases, Eleventh Revision (ICD-11) [6]. Diagnoses were originally recorded by the attending dentists using clinical terms (e.g., “caries,” “pulpitis”). For this study, two trained researchers retrospectively mapped these clinical diagnoses to the corresponding ICD-11 categories independently. Disagreements were resolved by consensus with a third reviewer. The five categories are: 1) Diseases of hard tissues of teeth, including caries, residual root, residual crown, fracture, dental defect, etc.; 2) Diseases of pulp or periapical tissues, including pulpitis, pulp necrosis, periapical periodontitis, periapical abscess, etc.; 3) Periodontal disease, including periodontitis, periodontal abscess, pericoronitis, pericoronal abscess, gingivitis, etc.; 4) Disorders of oral mucosa, including oral ulcer, oral mucosal infection, etc.; 5) Other oral diseases, including impaction, occlusal interferences, post-extraction complications, etc. The number of cases in each disease category and for each specific diagnosis was calculated.
(3) The trends of various diseases and categories across different batches were analyzed.

### Statistical analysis

Data analysis was performed using SPSS 25.0 software. A *P* value of < 0.05 was considered statistically significant.

For measurement data (age), normality of distribution was assessed using the Shapiro‑Wilk test, and homogeneity of variances was assessed using Levene’s test. Since both assumptions were met, pairwise independent-samples t‑tests were performed for age comparisons among the three batches. Measurement data were expressed as mean ± standard deviation (x̅ ± s).

Enumeration data included patients’ gender (male/female), the five categories of oral diseases (diseases of hard tissues of teeth, diseases of pulp or periapical tissues, periodontal disease, disorders of oral mucosa, and other oral diseases). These data were expressed as frequencies and percentages, and comparisons between groups were performed using the chi-square (*χ*²) test. The Cochran−Armitage test for trend was used to evaluate changes in proportions across the three batches. Results of the Cochran−Armitage test are reported as “*P* for trend”, while results of the chi‑square test are reported as “*P*”.

To adjust for multiple comparisons, Bonferroni correction was applied to all pairwise comparisons, including both age (independent-samples t-tests) and disease proportions (chi-square tests), with statistical significance set at *P* < 0.0167 (0.05/3). For comparisons that showed statistically significant differences after Bonferroni correction, effect sizes (Cramér’s V) and 95% confidence intervals for the differences in proportions were calculated to quantify the magnitude of the observed differences.

### Patient and public involvement

No patients or members of the public were involved in the design, conduct, reporting, or dissemination plans of this research.

### Ethics statement

This retrospective analysis of anonymized medical records was conducted in accordance with the Declaration of Helsinki. The study protocol was reviewed and granted an exemption from formal ethical approval by the Ethics Committee of the 940th Hospital of Joint Logistic Support Force of Chinese People’s Liberation Army (No reference number was assigned for this exemption). The exemption was granted because the study involved a retrospective analysis of medical records with no interaction with or intervention on human subjects. The data were collected from the Chinese level‑II hospital operating under MONUSCO, and data access and handling complied with the hospital’s data governance policies and relevant UN regulations for medical information within peacekeeping missions. Informed consent was waived because the study involved no more than minimal risk to participants, all data were fully de‑identified prior to analysis, and it was not feasible to obtain consent from all individuals given the retrospective nature and the multinational composition of the patient population.

## Results

### Patient demographics and backgrounds at the oral outpatient clinic

The demographics of patients attending the general outpatient departments and the oral outpatient clinic at the Chinese level-II hospital across Batches 23, 25, and 26 are shown in Table 1. There was no statistically significant difference in the age distribution of patients receiving oral outpatient services among Batches 23, 25, and 26 after Bonferroni correction (Batch 23 vs. 25: *P* = 0.017; Batch 23 vs. 26: *P* = 0.602; Batch 25 vs. 26: *P* = 0.074). Notably, the proportion of female patients in the oral outpatient clinic was significantly higher in Batch 25 than in Batches 23 and 26 (*χ*^2^ = 29.809, *P* < 0.001).

**Table 1:** Demographics of patients attending the general outpatient departments and the oral outpatient clinic across Batches 23, 25, and 26.

	Total outpatient visits, n	Age (years), mean ± SD	Oral outpatient visits, n	Age (years), mean ± SD	Female patients, n (%)
Batch 23	924	36.08 ± 7.58	205	34.88 ± 6.56	0.98%
Batch 25	726	38.63 ± 8.88	154	36.71 ± 7.54	11.04%
Batch 26	811	38.67 ± 9.37	206	35.25 ± 7.78	2.91%

n, number of observations; SD, standard deviation. No statistically significant differences in age were observed across batches after Bonferroni correction (adjusted α = 0.0167). The proportion of female patients was significantly higher in Batch 25 than in Batches 23 and 26 (*χ*² = 29.809, *P* < 0.001).

### Distribution of disease spectrum among patients of the oral outpatient clinic

The detailed distribution of oral diseases across the three batches is presented in Table 2. Overall, diseases of hard tissues of teeth and diseases of pulp or periapical tissues were the most frequent categories in all batches, while disorders of oral mucosa were the least frequent.

**Table 2:** Distribution of oral diseases among oral outpatient visits across Batches 23, 25, and 26.

	Disease type	Batch 23 (n = 205)	Batch 25 (n = 154)	Batch 26 (n = 206)
Diseases of hard tissues of teeth	Caries	24	30	62
	Residual root	8	10	16
	Residual crown	12	6	14
	Fracture	5	4	2
	Teeth defect	2	2	3
	Root resorption	1	0	0
	Dentin hypersensitivity	1	0	1
	Cracked tooth	0	2	0
Diseases of pulp or periapical tissues	Pulpitis	48	51	43
	Periapical periodontitis	32	18	25
	Pulp necrosis	10	1	10
	Periapical abscess	6	0	3
Periodontal disease	Gingivitis	20	0	2
	Periodontitis	1	3	7
	Pericoronitis	3	7	5
	Pericoronal abscess	3	0	0
	Periodontal abscess	0	7	0
	Peri-implantitis	2	0	0
	Tooth mobility	1	0	0
	Gingival atrophy	1	0	0
Disorders of oral mucosa	Oral ulcer	3	1	1
	Oral lichen planus	1	0	0
Other oral diseases	Impaction	12	1	3
	Dental extraction	0	1	3
	Post-dental extraction	1	3	0
	Tooth loss	0	1	0
	Occlusal interference	1	3	1
	Gingiva foreign body	0	1	0
	Trauma	0	1	0
	Food impacted	0	0	1
	Tooth elongation	0	0	1

n, number of observations. Disease categories were classified according to the International Classification of Diseases, 11th Revision (ICD-11). Specific diagnoses within each category are listed as recorded in the original clinical records.

### Analysis of the trends in disease categories across three batches of the oral outpatient visits

The oral outpatient visits in Batches 23, 25, and 26 ranked first in terms of number and proportion among all specialized outpatient consultations, with respective compositions of 22.19%, 21.21%, and 25.40%. The chi-square test showed no significant difference in the proportion of oral outpatient visits among the three batches (*χ*² = 4.298, *P* = 0.117). The Cochran−Armitage test for trend also indicated no significant linear trend across batches (*P* for trend = 0.204) (Table 3). The numbers and proportions of different categories of oral diseases across the three batches are shown in Table 4.

**Table 3:** Trends in the number and proportion of oral outpatient visits across Batches 23, 25, and 26.

	Batch 23	Batch 25	Batch 26	*χ* ^2^	*P*	*P for trend*
Total outpatient	924	726	811			
Oral outpatient	205 (22.19%)	154 (21.21%)	206 (25.40%)	4.298	0.117	0.204

*χ*², chi-square statistic; *P*, probability value. *P* for trend was calculated using the Cochran−Armitage test for trend. No statistically significant difference was observed in the proportion of oral outpatient visits across the three batches.

**Table 4:** Trends in the proportion of oral disease categories across Batches 23, 25, and 26.

Disease category	Batch 23 (n = 205)	Batch 25 (n = 154)	Batch 26 (n = 206)	*χ* ^2^	*P*	*P for trend*
Diseases of hard tissues of teeth	60 (29.27%) ^a^	55 (35.71%) ^a^	101 (49.03%) ^b^	17.558	<0.001	<0.001
Diseases of pulp or periapical tissues	96 (46.83%) ^a^	70 (45.45%) ^a^	81 (39.32%) ^a^	2.614	0.271	0.154
Periodontal disease	31 (15.12%) ^a^	17 (11.04%) ^a, b^	14 (6.80%) ^b^	7.292	0.026	0.008
Disorders of oral mucosa	4 (1.95%) ^a^	1 (0.65%) ^a^	1 (0.49%) ^a^	2.343	0.310	0.127
Other oral diseases	14 (6.83%) ^a^	11 (7.14%) ^a^	9 (4.37%) ^a^	1.574	0.455	0.360

n, number of observations; *χ*², chi-square statistic; *P*, probability value. *P* for trend was calculated using the Cochran−Armitage test for trend. Disease categories were classified according to the International Classification of Diseases, 11th Revision (ICD-11). Different superscript letters within the same row indicate statistically significant differences after Bonferroni correction for multiple comparisons (α = 0.0167).

Regarding cross‑batch comparisons: In Batch 23 and Batch 25, diseases of pulp or periapical tissues had the highest numbers and proportions, whereas in Batch 26, diseases of hard tissues of teeth had the highest numbers and proportions. In all three batches, disorders of oral mucosa had the lowest numbers and proportions.

Regarding trend analysis: From Batch 23 to Batches 25 and 26, there was an upward trend in the proportion of diseases of hard tissues of teeth, with a significantly higher proportion in Batch 26 compared to Batches 23 and 25. For example, the proportion was significantly higher in Batch 26 than in Batch 23 (*χ*² = 16.84, *P* < 0.001; Cramér’s V = 0.20, 95% CI for difference in proportions: 0.11–0.29). Similarly, it was significantly higher than in Batch 25 (*χ*² = 6.48, *P* = 0.011; Cramér’s V = 0.13, 95% CI for difference: 0.03–0.24). The proportion of diseases of pulp or periapical tissues decreased sequentially across the three batches, but the trend was not statistically significant. The proportion of periodontal disease declined, with the lowest proportion observed in Batch 26, which was significantly lower than that in Batch 23 (*χ*² = 7.31, *P* = 0.007; Cramér’s V = 0.13, 95% CI for difference in proportions: 0.02–0.14).

### Analysis of the trends of prevalent oral diseases across three batches

By selecting the diseases with the highest frequency in Batch 23, this study analyzed their trends across the three batches, ensuring that at least one disease from each disease category was included in the analysis. Specifically, caries, pulpitis, periapical periodontitis, and gingivitis were selected for further analysis. The trends of these four diseases across the three batches are presented in Table 5. From Batch 23 to Batches 25 and 26, the proportion of caries showed an upward trend, with a significantly higher proportion in Batch 26 compared to the Batch 23 (*χ*² = 21.44, *P* < 0.001; Cramér’s V = 0.23, 95% CI for difference in proportions: 0.12–0.25). Pulpitis had the highest proportion in Batch 25 but decreased significantly by Batch 26. The proportion of periapical periodontitis did not show significant changes across the three batches. The proportion of gingivitis showed a decreasing trend, with significantly higher proportions in Batch 23 compared to both Batch 25 (*χ*² = 18.76, *P* < 0.001; Cramér’s V = 0.23, 95% CI for difference: 0.06–0.14) and Batch 26 (*χ*² = 15.65, *P* < 0.001; Cramér’s V = 0.20, 95% CI for difference: 0.05–0.13).

**Table 5:** Trends in the proportion of frequently occurring oral diseases across Batches 23, 25, and 26.

Disease type	Batch 23 (n = 205)	Batch 25 (n = 154)	Batch 26 (n = 206)	*χ* ^2^	*P*	*P for trend*
Caries	24 (11.71%) ^a^	30 (19.48%) ^a, b^	62 (30.10%) ^b^	21.440	<0.001	<0.001
Pulpitis	48 (23.41%) ^a, b^	51 (33.12%) ^b^	43 (20.87%) ^a^	7.525	0.023	0.879
Periapical periodontitis	32 (15.61%) ^a^	18 (11.69%) ^a^	25 (12.14%) ^a^	1.540	0.463	0.255
Gingivitis	20(9.76%) ^a^	0 (0) ^b^	2(0.97%) ^b^	29.769	<0.001	<0.001

n, number of observations; *χ*², chi-square statistic; *P*, probability value. *P* for trend was calculated using the Cochran−Armitage test for trend. Different superscript letters within the same row indicate statistically significant differences after Bonferroni correction for multiple comparisons (α = 0.0167).

## Discussion

At this Chinese level-II hospital, oral outpatient visits represented the largest share of specialized outpatient consultations across the three analyzed batches and posed a significant challenge to the health of military and civilian personnel in the mission area. To our knowledge, this is among the first studies to examine temporal patterns of oral outpatient diagnoses in a peacekeeping hospital, and it also reflects the significant medical support challenges faced by the oral department of the Chinese level-II hospital.

The Chinese level-II hospital in the DRC primarily provides medical support for the military and civilian personnel of the MONUSCO southern sector. Since MONUSCO began to withdraw from the DRC gradually in 2018 [7], although the number of people supported by the Chinese level-II hospital has decreased and total outpatient visits have shown a downward trend, the number and proportion of oral outpatient visits from Batch 23 to Batches 25 and 26 have not declined significantly and have ranked first among all specialized outpatient clinics. Notably, the number of oral outpatient visits in Batch 26 reached the highest level among the three analyzed batches, placing greater demands on the hospital’s oral healthcare capacity.

This study shows that the proportion of diseases of hard tissues of teeth increased significantly across the three batches, reaching 49.03% in Batch 26 (*P* for trend < 0.001). Among these, caries had the highest number and proportion, and also showed an increasing trend (*P* for trend < 0.001). Caries is mainly associated with the metabolism of dietary sugars by oral bacteria [8], and its treatment usually involves removing the decayed tissue and filling the cavity [9]. The increase in consultation‑based caries burden observed in this study suggests that the Chinese level‑II hospital should prepare an adequate amount of dental restorative materials and preventive supplies, such as fluoride products.

There was no statistical difference in the proportion of diseases of pulp or periapical tissues among Batches 23, 25, and 26, and the trend was also not statistically significant; however, the proportion decreased sequentially across the batches. diseases of pulp or periapical tissues mainly include pulpitis and periapical periodontitis, both of which are the most common oral diseases at the Chinese level-II hospital. Pulpitis had the highest proportion in Batch 25, but it decreased significantly in Batch 26. There was no significant difference in the proportion of periapical periodontitis among the three batches. Dental caries is the most common risk factor for pulpitis [10], while periapical periodontitis is usually associated with the spread of infection from untreated pulpitis to the tissues surrounding the root tip of the tooth [11]. Dental trauma that exposes the pulp can also lead to pulpitis and periapical periodontitis. A small number of pulp or periapical diseases in the mission area are caused by trauma, and periodontal disease can also lead to periapical periodontitis. The treatment of diseases of pulp or periapical tissues usually involves root canal therapy, apical surgery, or pulp revascularization [12]. Good oral hygiene, regular examinations, and dietary modification — reducing the intake of high-sugar and high-acid foods — are key to preventing such diseases [13]. In Batch 26 at the Chinese level-II hospital, while the proportion of caries increased significantly, the proportion of pulpitis decreased. Although this simultaneous trend is noteworthy, this study cannot determine whether the decline in pulpitis is directly attributable to treatment effectiveness. Other factors, such as changes in diagnostic practices, population composition, or access to care, may also have contributed. Future prospective studies are needed to evaluate whether caries treatment is associated with a reduced risk of pulpitis.

The proportion of periodontal disease also showed a downward trend from Batch 23 to Batches 25 and 26, with the proportion of periodontal disease in Batch 26 being significantly lower than that in Batch 23. Gingivitis was the most prevalent periodontal disease in Batch 23, but its proportion decreased significantly in Batches 25 and 26. Periodontal disease is associated with dental plaque, tartar, and delayed treatment of caries, as well as genetic factors, immune status, and lifestyle habits. Poor oral hygiene, tobacco use, and irregular diet are recognized risk factors [14]. Treatment primarily involves tooth surface cleaning, including brushing, flossing, and rinsing, to remove plaque and tartar. For established periodontal disease, conventional treatments include gingival scaling, root planing, root surface filling, and periodontal surgery; severe cases such as periodontal abscesses may require surgical intervention [15,16]. Smoking cessation, alcohol restriction, and healthy lifestyles: sufficient sleep, balanced diet, and moderate exercise−are effective preventive measures [17,18].

Disorders of oral mucosa accounted for a relatively low proportion, with their proportions in Batches 25 and 26 both being below 1% and only one case reported in each. Given the very low number of consultations for disorders of oral mucosa, they may represent a lower priority for resource allocation compared to other disease categories in this specific hospital setting. In Batch 25, the proportion of female patients was significantly higher than that in Batches 23 and 26. However, there is no evidence to suggest a direct relationship between gender and the prevalence of oral diseases such as caries in the mission area [19]. Some studies have indicated that men are more prone to periodontal disease compared to women, mainly because men are more susceptible to certain pathogenic factors, such as smoking, poor oral hygiene habits, and inadequate oral health literacy [20].

Although the major troop‑contributing countries (e.g., Bangladesh, India, Nepal, and Pakistan) are relatively consistent across batches, and oral diseases develop over long periods closely linked to personal habits, the disease spectrum observed in the DRC mission area may offer only a preliminary reference for other peacekeeping missions, given differences in mission environments and personnel characteristics. This extrapolation should therefore be regarded as a hypothesis to be tested in future studies.

For MONUSCO itself, our findings have direct operational implications. The persistent high demand for dental care and the increasing proportion of caries across successive batches highlight the need for continuous monitoring of oral disease patterns. We recommend that future rotations implement pre-deployment oral health screening, regular in-mission dental check-ups, and sustained oral health education to reduce the burden of advanced diseases such as pulpitis and to improve the overall oral health readiness of peacekeeping personnel.

It is important to acknowledge that the study period partially overlapped with the COVID‑19 pandemic and associated lockdown measures. During lockdowns, access to routine dental care may have been restricted, which may have contributed to an underrepresentation of mild conditions (e.g., initial caries) and a relative increase in more advanced diseases (e.g., pulpitis). Conversely, when restrictions eased, previously accumulated unmet dental needs may have been associated with a surge of consultations for hard tissue diseases, as seen in Batch 26. Although we cannot precisely quantify the effect due to the retrospective design, we acknowledge that it likely influenced the observed trends. Future prospective studies should account for such external shocks.

### Limitations

This study has several limitations. First, this was a single‑center study conducted at one Chinese level‑II hospital in Bukavu, DRC, which may introduce selection bias as it included only patients who actively sought care at this facility. Whether the findings are applicable to other mission areas remains uncertain, and multicenter validation is needed. Second, the analysis was based on only three batches, with a relatively short observation period, which may not capture long‑term trends in oral disease patterns, and the sample size was relatively small. Third, we did not perform subgroup analyses (e.g., by age, sex, rank, mission type, or duration of service), and thus could not fully account for the heterogeneity of the peacekeeper population. Fourth, this study relied solely on clinical records from the Chinese level‑II hospital and did not include data or perspectives from local Congolese healthcare institutions or doctors. Fifth, this study did not collect or analyze several key determinants of oral diseases, including nationality, duration of deployment, lifestyle factors (diet, smoking, alcohol), underlying stomatological conditions, or prior access to dental care. These factors were mentioned in the Discussion based on the literature, but due to the retrospective nature of the data, we could not verify their actual impact. Sixth, the analysis is visit‑based rather than person‑based, which limits inference about individual‑level disease burden and may be subject to selection bias, as individuals with oral conditions who did not present for care were not captured. Seventh, disease classification relied on routine clinical records and may be subject to diagnostic or coding misclassification, representing a potential source of diagnostic bias in the absence of independent verification or calibration among clinicians. Eighth, exclusion of Batch 24 due to incomplete records may have affected temporal continuity, and changes in mission conditions across batches may have influenced healthcare‑seeking behavior and disease patterns, although these factors were not directly measured. Ninth, this study did not account for potential confounders such as changes in personnel composition, mission conditions, staffing or diagnostic capacity, or population size across the three batches. As a result, the observed trends in disease proportions should be interpreted as descriptive findings rather than causal or risk‑adjusted epidemiological changes. The retrospective design inherently limits causal inference, and all findings should be interpreted as descriptive associations rather than causal relationships. Future studies with detailed data on these contextual factors are needed to confirm whether the observed trends reflect true changes in disease burden.

## In conclusion

Oral diseases accounted for the largest proportion of specialized outpatient consultations at the Chinese level-II hospital across the three analyzed batches. The observed decline in periodontal disease and pulpitis in later batches, along with the increase in caries, suggests that continuous oral health education and ensuring adequate supplies of restorative materials should be priorities for future rotations. However, the marked increase in hard tissue diseases, particularly caries, in Batch 26 highlights a key area requiring focused attention and resource allocation at the Chinese level-II hospital. During the mission preparation stage, it is essential to increase the supply of materials related to caries treatment and prevention. These findings may help inform future assessments of dental staffing and material requirements in comparable peacekeeping hospitals. Of note, the latest United Nations requirements for oral services in the Chinese level-II hospitals have already included an additional dental technician, which is consistent with our view [3]. Furthermore, given that the major troop-contributing countries are relatively consistent across missions and that oral diseases develop over long periods closely linked to personal habits, the findings may provide preliminary operational insights for peacekeeping hospitals operating in similar settings. However, validation in other missions and contexts is required before the findings can be generalized more broadly.

## Supporting information

S1 Data Minimal anonymized individual-level dataset underlying the main findings of this study. This file contains the anonymized individual‑level clinical data for all oral outpatient visits included in the analysis (after excluding repeated visits for the same disease). All personal identifiers have been removed to protect patient privacy.
(XLSX)
